# Mortality Predictors of Adult Meningeal Tuberculosis: Analysis from a Tertiary Center Using the Lancet Consensus Scoring System

**DOI:** 10.7759/cureus.101052

**Published:** 2026-01-07

**Authors:** Pratiksha Kamboj, Yogendra P Mathuria, Pratima Gupta, Ruchi Dua, Deepika Chakraborty, Binal Mangroliya, Sindura P

**Affiliations:** 1 Microbiology, All India Institute of Medical Sciences, Rishikesh, Rishikesh, IND; 2 Pulmonary Medicine, All India Institute of Medical Sciences, Rishikesh, Rishikesh, IND

**Keywords:** human immunodeficiency virus, lancet consensus scoring system, morbidity, mortality, mycobacterium tuberculosis, tuberculous meningitis

## Abstract

Introduction: Compared with other types of central nervous system tuberculosis (TB), meningeal TB has a greater fatality rate. However, data on mortality predictors are limited. This study aimed to determine predictors of mortality in patients with meningeal TBM.

Study design: This was a hybrid retrospective-prospective (ambispective) study, in which data were collected retrospectively for patients admitted between January 2019 and December 2020 and prospectively for patients admitted between January 2021 and March 2022.

Materials and methods: We conducted an ambispective cohort study of 132 adults (>18 years) with suspected meningitis admitted to the All India Institute of Medical Sciences, Rishikesh, India, between January 2019 and March 2022. Patients were classified as having definite, probable, or possible tuberculous meningitis (TBM) using the Lancet Consensus case definition. The primary outcome was in-hospital mortality. Univariable and multivariable Cox proportional-hazards models were used to identify predictors.

Results: Of the 132 patients enrolled in the study, 66 were classified as having definitive TBM, 12 as probable TBM, 21 as possible TBM, and 33 as non-TBM. The overall mortality rate among patients with any form of TBM (definitive, probable, or possible) was 37.87% (25 deaths), with the highest mortality observed in the definitive TBM group, accounting for 32.3% of deaths (21 cases). Fever, altered sensorium, high serum neutrophil count, low serum lymphocyte count, high CSF lymphocyte count, and low CSF neutrophil count were significantly associated (p<0.05) with the variable definitive TBM as a predictor of mortality in our study.

Conclusions: Simple hematological and CSF parameters can help stratify risk in TBM. These markers may guide early clinical decisions and closer monitoring.

## Introduction

Globally, 1.6 million people (including 187,000 HIV-positive individuals) died from tuberculosis (TB) in 2021. In terms of infectious agent-related mortality, TB ranks second globally and is the most prevalent infectious disease to cause death, following COVID-19 [1].

An estimated 10.6 million people globally contracted TB in 2021. In 2021, 54% of HIV-negative individuals who died from TB globally were men, 32% were women, and 14% were children under the age of fifteen. The greater percentage of children in comparison with their anticipated case share of 11% implies less access to diagnosis and treatment. A total of 51% of the HIV-positive individuals who died from TB were men, 38% were women, and 11% were children [1]. A direct national incidence estimate for adult tuberculous meningitis (TBM) in India is unavailable, owing to underdiagnosis, the absence of routine surveillance for central nervous system TB, and dependence on modeled estimates derived from overall TB burden data.

One extreme of extrapulmonary TB that affects the central nervous system, TBM, remains one of the most challenging among the whole spectrum of TB. Although estimates of the burden of TBM locally or globally are not readily available, different studies conducted over time have reported different proportions of cases along with variability in proportions across locations and overall TB prevalence [2]. Adult TBM patients have a mortality rate between 30% and 60%, and more than 50% of survivors have experienced neurological aftereffects [3]. The nonspecific clinical picture and low sensitivity of microbiological confirmation testing further complicate the diagnosis of TB [4]. 

Meningeal TB has a higher death rate than other forms of TB affecting the central nervous system [5]. Data on mortality predictors, however, are scarce. In this study, the Lancet Consensus Scoring System was used to categorize definite, probable, and possible TBM [6].

## Materials and methods

This study was conducted at a tertiary care teaching hospital and an institute of national importance located in Rishikesh, Uttarakhand, India. The hospital serves as a major referral center for the Himalayan region, providing specialized care for infectious diseases, including TBM. The study aimed to identify clinical, laboratory, and radiological predictors of mortality among adult patients diagnosed with TBM.

A cross-sectional observational study design was employed. Ethical approval for the study was obtained from the Institutional Ethics Committee of the All India Institute of Medical Sciences, Rishikesh, India, with the reference number 109/IEC/PGM/2021 prior to data collection. All procedures adhered to the principles outlined in the Declaration of Helsinki. 

The study included all adult patients aged 18 years and above who were admitted with suspected meningitis between January 1, 2019, and March 31, 2022. Patients were eligible if they were ≥18 years old, presented with clinical suspicion of meningitis, and met the diagnostic criteria for definite, probable, or possible TBM based on the Lancet Consensus Scoring System [6]. Patients with confirmed alternative causes of meningitis, including bacterial, viral, fungal, or parasitic etiologies; those with known malignancies or lymphoma; and those who declined participation or for whom consent could not be obtained were excluded. CSF samples were obtained via lumbar puncture as part of routine evaluation and were subjected to biochemical analysis (protein, glucose, and cell counts), microbiological testing (Ziehl-Neelsen staining, cartridge-based nucleic acid amplification test (CBNAAT), and culture for *Mycobacterium tuberculosis*), and additional investigations such as CT or MRI of the brain and systemic evaluation when indicated.

Diagnostic classification followed the Lancet consensus scoring system, categorizing patients as definite TBM when microbiological confirmation was present, probable TBM when the score was >10 without imaging or >12 with imaging, possible TBM when the score ranged from 6 to 9 without imaging or 6 to 11 with imaging, and not TBM when scores were <6 or an alternative diagnosis was identified (Table 1) [6]. The primary outcome assessed was in-hospital mortality, and data on demographic details, clinical features, laboratory parameters, imaging findings, and comorbidities were collected for analysis. Information was retrieved from the hospital electronic medical records and laboratory information system using standardized case-report forms to maintain uniformity. Statistical analysis was performed using appropriate tests based on variable type and distribution, with categorical variables summarized as frequencies and percentages and compared between survivors and non-survivors using the chi-square test or Fisher’s exact test when cell counts were <5. Continuous variables were checked for normality, and because many were non-normally distributed, the Wilcoxon-Mann-Whitney U test was used for comparisons. A p-value < 0.05 was considered statistically significant, and multivariable regression analysis was planned where the sample size was sufficient to identify independent predictors of mortality.

**Table 1:** The Lancet Consensus Scoring System CSF: cerebrospinal fluid; TST: tuberculin skin test; IGRA: interferon-gamma release assay; CNS: central nervous system; CT: computed tomography scan; MRI: magnetic resonance imaging; NAAT: nucleic acid amplification test; AFB: acid-fast basilli Source: [6]

Clinical Criteria	Score (Maximum Category Score = 6)
Symptom duration of more than five days	4
Systemic symptoms suggestive of tuberculosis (one or more of the following): weight loss (or poor weight gain in children), night sweats, or persistent cough for more than 2 weeks	2
History of recent (within the past year) close contact with an individual with pulmonary tuberculosis or a positive TST or IGRA (only in children	2
Focal neurological deficit (excluding cranial nerve palsies)	1
Cranial nerve palsy	1
Altered consciousness	1
CSF criteria	(Maximum category score = 4)
Clear appearance	1
Cells: 10–500 per μl	1
Lymphocytic predominance (> 50%)	1
Protein concentration greater than 1 g/L	1
CSF to plasma glucose ratio of less than 50% or an absolute CSF glucose concentration less than 2·2 mmol/L	1
Cerebral imaging criteria	(Maximum category score = 6)
Hydrocephalus	1
Basal meningeal enhancement	2
Tuberculoma	2
Infarct	1
Pre-contrast basal hyperdensity	2
Evidence of tuberculosis elsewhere	(Maximum category score = 4)
Chest radiograph suggestive of active tuberculosis: signs of tuberculosis = 2; miliary tuberculosis = 4	2/4
CT/ MRI/ ultrasound evidence for tuberculosis outside the CNS	2
AFB identified or *Mycobacterium tuberculosis* cultured from another source—i.e., sputum, lymph node, gastric washing, urine, blood culture	4
Positive commercial *Mycobacterium tuberculosis *NAAT from extra-neural specimen	4

## Results

Of 132 patients admitted with suspected meningitis, 99 fulfilled the Lancet Consensus Criteria for TBM (definite: 66, probable: 12, possible: 21), and 33 had no TBM.

A total of 31 patients died due to the meningitis. The overall mortality rate among patients with any form of TBM (definite, probable, or possible) was 37.87% (25 deaths), with the definite TBM group showing the highest mortality, representing 32.3% of the deaths (21 cases).

The median age with interquartile range of these patients was 34.50 years, and the male-to-female ratio was 1:1.06 (Table 2).

**Table 2:** Age and gender distribution SD: standard deviation; Min-Max: minimum to maximum

Basic details	Mean ± SD || Median (IQR) || Min-Max || Frequency (%)	Total
Age (years)	38.55 ± 17.13 || 34.50 (23.00-50.00) || 18.00 - 90.00	
Age	Frequency (%)	
18-40 years	80 (60.6%)	132
41-60 years	35 (26.5%)	
>60 years	17 (12.9%)	
Gender		
Male	63 (47.7%)	132
Female	69 (52.3%)	

Our study revealed fever, altered sensorium, and serum and CSF biochemical analysis findings as predictors of mortality in any TBM (definite, probable, and possible patients) (Table 3). The factors that were not associated with mortality included the clinical features of age, sex, vomiting, headache, neck stiffness, seizure, behavioral disturbances, any predisposing factor, and microbiological investigation via multiplex polymerase chain reaction (PCR) (Table 3). Multiple logistic regression revealed fever (p=0.007), altered sensorium (p=0.001), increased serum neutrophil count (p=0.010), reduced serum lymphocyte count (p=0.002), increased CSF lymphocyte count (p=0.030), and reduced CSF neutrophil count (p=0.016) as significant risk factors for mortality in TBM patients (Table 3).

**Table 3:** Association between the parameters and mortality ***Significant at p<0.05; ^1^Wilcoxon-Mann‒Whitney U Test; ^2^chi-squared test, ^3^Fisher's exact test ZN: Ziehl–Neelson stain; CBNAAT: cartridge-based nucleic acid amplification test; AFB: acid-fast bacilli; TBM: tuberculous meningitis; CSF: cerebrospinal fluid

Parameters	Any TBM		Test Value	p-value
	Yes (n=31)	No (n=101)		
Age			1.071	0.585^2^
18-40 years	20 (64.5%)	60 (60.0%)		
41-60 years	6 (19.4%)	29 (28.7%)		
>60 years	5 (16.1%)	12 (12.0%)		
Gender			0.202	0.653^2^
Male	16 (51.6%)	47 (47.0%)		
Female	15 (48.4%)	54 (54.0%)		
Presentation: Fever (Present)***	27 (87.1%)	61 (61.0%)	7.309	0.007^2^
Presentation: Headache (Present)	16 (51.6%)	44 (44.0%)	0.552	0.457^2^
Presentation: Vomiting (Present)	13 (41.9%)	35 (35.0%)	0.490	0.484^2^
Presentation: Altered sensorium (Present)***	26 (83.9%)	51 (51.0%)	10.553	0.001^2^
Presentation: Neck rigidity (Present)	4 (12.9%)	16 (16.0%)	0.175	0.782^3^
Presentation: Duration of symptom >5 Days (Present)	24 (77.4%)	82 (82.0%)	0.322	0.571^2^
Any pre-disposing factor (Present)	16 (51.6%)	47 (47.0%)	0.202	0.653^2^
Imaging suggestive of TBM (Yes)	7 (50.0%)	37 (71.2%)	2.221	0.201^3^
Neutrophils (%)	83.95 ± 13.12	76.94 ± 12.87	794.000	0.010^1^
Lymphocytes (%)***	7.44 ± 3.97	14.93 ± 9.96	298.500	0.002^1^
CSF: Sugar (mg/dL)	48.63 ± 41.95	54.64 ± 38.69	1282.500	0.169^1^
CSF: Protein (mg/dL)	227.97 ± 200.97	177.39 ± 169.96	1695.000	0.243^1^
CSF: Leucocytes (Present)	19 (76.0%)	70 (73.7%)	0.055	0.814^2^
CSF: Polymorphs (%)***	48.37 ± 35.12	28.39 ± 30.50	903.000	0.016^1^
CSF: Lymphocytes (%)***	53.70 ± 36.34	71.86 ± 30.74	486.500	0.030^1^
ZN stain (Positive)	2 (6.5%)	1 (1.0%)	3.143	0.139^3^
CBNAAT (Positive)	18 (64.3%)	44 (44.4%)	3.439	0.064^2^
Rifampicin resistance (Positive)	3 (12.0%)	3 (3.6%)	2.631	0.132^3^
AFB culture (Positive)	1 (25.0%)	3 (12.0%)	0.490	0.467^3^

Out of 132 patients, 66 were diagnosed with TBM. Among these diagnosed patients, 39 showed improvement after treatment and were discharged. Sixteen patients died before treatment could be initiated, while five died following the start of treatment. Additionally, six diagnosed patients were lost to follow-up (Table 4).

**Table 4:** Outcome TBM: tuberculous meningitis

Outcome	All Suspects (n=132)
Definite TBM	66
Discharged	39
Died before treatment	16
Died after treatment	5
Lost to follow-up	6

## Discussion

This study revealed almost equal numbers of male and female patients. The male-to-female ratio was 0.91:1. This is in contrast with the data published by Kaur et al. [7], who reported a greater predominance of cases in males than in females. One possible explanation for this difference in gender distribution could be the relatively higher literacy rate among females in Uttarakhand compared to other Indian states. Increased female literacy in Uttarakhand might lead to better awareness, earlier healthcare-seeking behavior, and improved diagnosis in women, which could influence the gender ratio observed in TBM cases in the state [8]. Additionally, sociocultural factors linked with education may contribute to enhanced access to medical care for women in this region, potentially balancing the male predominance seen elsewhere in India. These factors combined may explain the differing epidemiological patterns between Uttarakhand and other regions, as noted by Kaur et al. [7].

The overall mortality rate of patients with any TBM (definite, probable, possible) was 25 (37.87%), of which the mortality rate of patients with definite TBM was the highest, i.e., 21 (32.3%). This was in accordance with a study conducted in North India, which reported a mortality rate of 43.63%.

In this study altered sensorium was identified as a significant predictor of mortality in our study, consistent with findings from a similar multicentric study worldwide by Wen et al., an Indian multi-center study by Gupta et al., and the Iranian study by Maleki Rad et al., where it also emerged as an important clinical factor associated with increased risk of death in tuberculous meningitis [9-11]. These findings underscore the increased susceptibility of affected individuals and highlight the urgent need for prompt recognition and early intervention, particularly in cases presenting with altered mental status, to improve clinical outcomes.

In our study, fever was a predictor of mortality. A similar hospital-based study in Indonesia by van Laarhoven et al. revealed fever, focal neurological signs, and altered sensorium as predictors of mortality [12]. Our study demonstrated that high CSF lymphocytes and low CSF neutrophils are predictors of mortality. A hospital-based study in South Africa by Nonkala et al. and a multicentric Iranian study by Maleki Rad et al. reported similar findings [11, 13]. Elevated neutrophil levels in the CSF have shown a positive association with survival rates in TBI patients, and in adults with TBM, higher CSF neutrophil counts have also been correlated with the occurrence of cerebral infarctions as identified by head MRI studies [14]. In our study, high serum neutrophil counts and low serum lymphocyte counts were also predictors of mortality. Similar findings were reported in a hospital in China by Gu et al. [15]. Our study revealed a very high mortality rate in definite TBM patients, i.e., 38% (n=21). A systematic review by Seid et al. aligned with our study that reported a 20.03% case fatality rate during illness [16]. Patients with confirmed tubercle bacilli in CSF often have a higher bacterial burden, leading to greater neurological injury and poorer outcomes. Disturbances in consciousness and severe neurological deficits are common and significantly increase the risk of death [11]. Although various scoring systems are available for the diagnosis of tuberculous meningitis, the Thwaites, Marais, and Lancet Consensus Scoring Systems are the most well-established. In our study, we utilized the Lancet Consensus Scoring System for the diagnosis of TBM. A multicentric study by Sulaiman et al. showed that the Thwaites Scoring System had poor diagnostic accuracy, whereas the Lancet Scoring System demonstrated fair accuracy for diagnosing tuberculous meningitis [17]. The Thwaites Diagnostic Score is simple to use and effectively differentiates TB meningitis from bacterial meningitis with good accuracy. In contrast, the Lancet Consensus Score is more detailed, may require specialist input, and provides good accuracy in distinguishing TB meningitis from other causes [18].

The strengths of this study include its ambispective design, the use of standardized diagnostic criteria, and adjustment for clinical severity. However, several limitations should be acknowledged, including the single-center setting, a relatively small sample size that may have limited the stability of multivariable analyses, potential referral bias, and incomplete long-term follow-up. In addition, detailed information on treatment adherence and neurosurgical interventions was unavailable, which may have influenced the observed outcomes.

## Conclusions

Simple, routinely available hematologic and CSF parameters may serve as valuable tools for mortality risk stratification in TBM, especially in resource-limited settings. Patients identified as high-risk could benefit from closer monitoring, adjunctive therapies, and timely escalation of care. Future multicenter studies with standardized follow-up and biomarker validation are warranted. Incorporating host immune markers and genomic diagnostics may further enhance risk prediction and guide individualized management in TBM. The pronounced mortality rate within the adult TBM population necessitates a proactive approach to patient management.

By maintaining strong clinical suspicion for specific symptoms and actively considering prognostic determinants, clinicians can work toward minimizing the impact of this condition on patients’ lives.
